# Supplementary material for: Mammalian histones facilitate antimicrobial synergy by disrupting the bacterial proton gradient and chromosome organization
Source: Nat Commun. 2020 Aug 4;11:3888. doi: 10.1038/s41467-020-17699-z (PMC7403156; doi:10.1038/s41467-020-17699-z)
Supplement: Supplementary file 1 — Supplementary Information [file 41467_2020_17699_MOESM1_ESM.pdf]

Mammalian histones facilitate antimicrobial synergy by disrupting the bacterial proton gradient and chromosome organization, Doolin et al.

Supplementary Information

Supplementary Table 1
Supplementary Figures 1-5
Source Data

SUPPLEMENTARY TABLE 1

Gene	Function	10/0	50/0	100/0
wza	colanic acid export protein; outer membrane auxillary lipoprotein	2.9	43.3	72.8
wzb	colanic acid production protein-tyrosine-phosphatase; Wzc-P dephosphorylase	3.9	40.2	70.2
wcaE	putative glycosyl transferase	2.5	34.6	55.7
wcaG	bifunctional GDP-fucose synthetase: GDP-4-dehydro-6-deoxy-D-mannose epimerase/ GDP-4-dehydro-6-L-deoxygalactose reductase	3.1	29.8	49.8
bdm	biofilm-dependent modulation protein	4.0	35.3	49.6
yjbE	extracellular polysaccharide production threonine-rich protein	2.8	29.5	47.5
yjbF	extracellular polysaccharide production lipoprotein	2.9	25.8	44.0
wcaA	putative glycosyl transferase	2.1	23.3	39.7
rprA	Null	3.4	23.1	39.5
wcaD	putative colanic acid polymerase	2.2	23.3	37.6
gmd	GDP-D-mannose dehydratase, NAD(P)-binding	2.4	22.3	36.6
wzc	colanic acid production tyrosine-protein kinase; autokinase; Ugd phosphorylase	2.1	21.6	36.3
yjbG	extracellular polysaccharide export OMA protein	2.2	20.5	34.9
wcaF	putative acyl transferase	1.6	19.5	32.2
ymgI	uncharacterized protein	1.0	25.1	32.0
osmB	osmotically and stress inducible lipoprotein	4.2	24.5	30.6
wcaI	putative glycosyl transferase	2.3	18.5	30.2
cpsG	phosphomannomutase	1.9	19.4	30.2
wcaH	GDP-mannose mannosyl hydrolase	1.8	16.1	26.4
wcaJ	colanic biosynthesis UDP-glucose lipid carrier transferase	2.3	16.5	25.2
cpsB	mannose-1-phosphate guanyltransferase	2.1	15.2	24.7
rcsA	transcriptional regulator of colanic acid capsular biosynthesis	1.9	14.7	21.6
ymgG	UPF0757 family protein	2.9	17.2	21.6
wcaC	putative glycosyl transferase	1.8	12.4	20.4
wzxC	putative colanic acid exporter	2.0	12.8	18.5
ymgD	periplasmic protein, HdeA structural homolog	2.5	14.8	18.2
wcaB	putative acyl transferase	1.4	9.7	16.9
wcaK	colanic acid biosynthesis protein	1.6	11.3	14.8
ivy	inhibitor of c-type lysozyme, periplasmic	1.8	10.6	14.7
yjbH	DUF940 family extracellular polysaccharide protein	1.4	8.9	14.3
ygaC	uncharacterized protein	2.1	10.1	14.1
mliC	inhibitor of c-type lysozyme, membrane-bound; putative lipoprotein	1.5	7.0	10.2
osmY	salt-inducible putative ABC transporter periplasmic binding protein	1.6	6.6	9.4
ugd	UDP-glucose 6-dehydrogenase	1.2	6.6	8.9
ygdI	DUF903 family verified lipoprotein	1.6	6.0	8.2
ypeC	DUF2502 family putative periplasmic protein	1.5	5.3	8.2
ytjA	uncharacterized protein	1.4	5.5	8.1
ycfJ	uncharacterized protein	1.4	5.0	7.7
ybgS	putative periplasmic protein	1.2	5.2	7.2
wcaL	putative glycosyl transferase	1.3	5.4	7.1
yaiY	DUF2755 family inner membrane protein	1.4	4.8	7.0
iraM	RpoS stabilzer during Mg starvation, anti-RssB factor	1.1	5.1	6.8
ydeI	hydrogen peroxide resistance OB fold protein; putative periplasmic protein	0.9	5.6	6.6
ecpR	putative transcriptional regulator for the ecp operon	1.2	4.8	6.2
yghA	putative oxidoreductase	1.1	4.1	6.0
ycfT	inner membrane protein	1.1	3.7	5.9
katE	catalase HPII, heme d-containing	1.2	4.2	5.8
degP	serine endoprotease (protease Do), membrane-associated	1.2	3.6	5.3
osmC	lipoyl-dependent Cys-based peroxidase, hydroperoxide resistance; salt-shock inducible membrane protein; peroxiredoxin	1.4	3.9	5.1
yiaD	multicopy suppressor of bamB; outer membrane lipoprotein	1.5	4.3	5.1
yegS	phosphatidylglycerol kinase, metal-dependent	1.2	4.0	5.0
sra	stationary-phase-induced ribosome-associated protein	1.3	3.9	5.0
ecpB	ECP production pilus chaperone	1.2	2.9	4.8
ypfG	DUF1176 family protein	1.2	3.4	4.7
yohP	uncharacterized protein	2.0	3.1	4.7
yiaB	YiaAB family inner membrane protein	0.7	3.1	4.6
Gene	**Function**	**10/0**	**50/0**	**100/0**
hslJ	heat-inducible lipoprotein involved in novobiocin resistance	1.3	3.9	4.6
ecpA	ECP pilin	1.1	3.3	4.6
yjdP	putative periplasmic protein	1.3	3.5	4.5
bax	putative glucosaminidase	1.5	3.9	4.5

yajI	putative lipoprotein	1.4	3.2	4.4
loiP	Phe-Phe periplasmic metalloprotease, OM lipoprotein; low salt-inducible; Era-binding heat shock protein	1.2	3.3	4.3
spy	periplasmic ATP-independent protein refolding chaperone, stress-induced	0.8	2.7	4.3
osmE	osmotically-inducible lipoprotein	1.4	3.3	4.2
yjbT	putative periplasmic protein	1.3	3.0	4.1
ybjP	lipoprotein	1.2	3.4	4.0
ytfK	DUF1107 family protein	1.1	2.8	3.9
ysaB	uncharacterized protein	1.3	3.6	3.9
galP	D-galactose transporter	1.2	3.0	3.8
rcnB	periplasmic modulator of Ni and Co efflux	1.3	3.4	3.7
ybaY	outer membrane lipoprotein	1.1	2.7	3.7
omrB	Null	1.1	2.4	3.7
omrA	Null	1.0	2.5	3.6
eco	ecotin, a serine protease inhibitor	1.2	2.8	3.5
stpA	DNA binding protein, nucleoid-associated	1.6	3.8	3.4
wcaM	colanic acid biosynthesis protein	1.2	3.1	3.4
ygdR	DUF903 family verified lipoprotein	1.1	2.7	3.3
ydeT'	pseudogene	0.8	3.0	3.3
ygaM	putative membrane-anchored DUF883 family ribosome-binding protein	1.1	2.2	3.3
glsA	glutaminase 1	1.5	2.0	3.2
yhbO	stress-resistance protein	1.0	2.3	3.2
yggE	oxidative stress defense protein	1.2	2.6	3.1
ybiH	DUF1956 domain-containing tetR family putative transcriptional regulator	1.3	2.0	3.1
yodB	cytochrome b561 homolog	1.2	2.6	3.1
ygiM	SH3 domain protein	1.1	2.4	3.0
ydeQ	putative fimbrial-like adhesin protein	1.3	2.1	3.0
ybhG	putative membrane fusion protein (MFP) component of efflux pump, membrane anchor	1.4	1.8	3.0
otsB	trehalose-6-phosphate phosphatase, biosynthetic	1.3	2.2	2.9
yjbJ	stress-induced protein, UPF0337 family	1.4	2.4	2.9
ybfA	DUF2517 family protein	1.2	1.5	2.9
ybdG	mechanosensitive channel protein, miniconductance	1.2	2.3	2.9
yehX	putative ABC transporter ATPase	1.4	1.7	2.8
fepA	ferrienterobactin outer membrane transporter	1.0	2.2	2.8
gmr	cyclic-di-GMP phosphodiesterase; csgD regulator; modulator of RNase II stability	1.0	2.2	2.8
ybdZ	stimulator of EntF adenylation activity, MbtH-like	1.0	2.2	2.7
entE	2,3-dihydroxybenzoate-AMP ligase component of enterobactin synthase multienzyme complex	1.0	2.0	2.7
yfeY	RpoE-regulated lipoprotein	1.1	2.0	2.7
yceB	lipoprotein, DUF1439 family	1.1	2.2	2.7
ydeS	putative fimbrial-like adhesin protein	1.2	1.8	2.6
caiD	carnitinyl-CoA dehydratase	1.4	2.3	2.6

Supplemental Table 1. Fold-change in mRNA transcript in the top 100 genes up-regulated in E. coli following a 30-minute treatment with H2A. The 10/0, 50/0, and 100/0 labels indicate comparison between 10, 50, or 100 µg/mL H2A and untreated cells. Values are the average of three independent experiments.

SUPPLEMENTARY FIGURES

Supplementary Figure 1

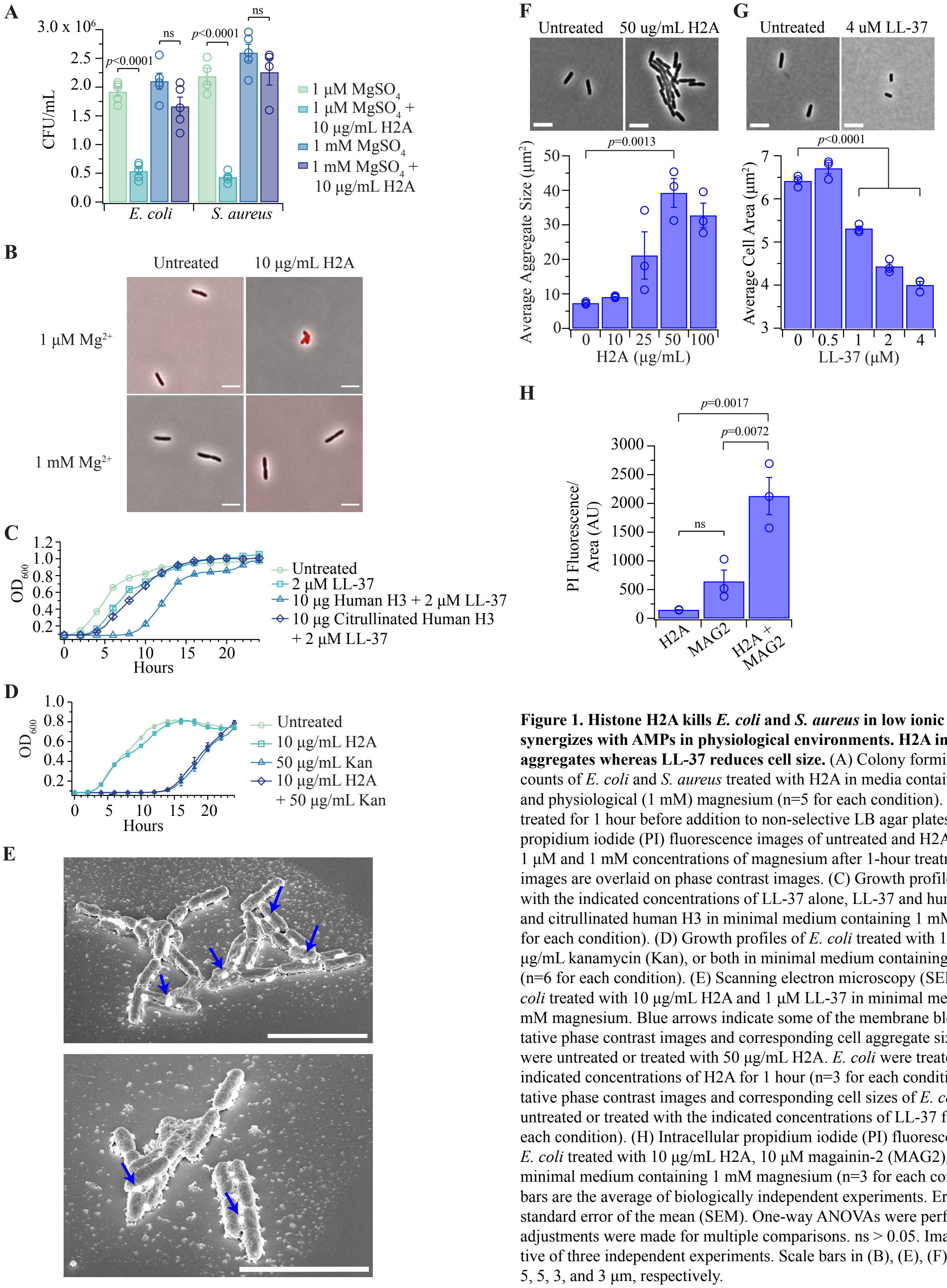

Figure 1. Histone H2A kills *E. coli* and *S. aureus* in low ionic environments and synergizes with AMPs in physiological environments. H2A induces cell aggregates whereas LL-37 reduces cell size. (A) Colony forming unit (CFU) counts of *E. coli* and *S. aureus* treated with H2A in media containing low (1 µM) and physiological (1 mM) magnesium (n=5 for each condition). Bacteria were treated for 1 hour before addition to non-selective LB agar plates. (B) Intracellular propidium iodide (PI) fluorescence images of untreated and H2A-treated *E. coli* in 1 µM and 1 mM concentrations of magnesium after 1-hour treatment. Fluorescence images are overlaid on phase contrast images. (C) Growth profiles of *E. coli* treated with the indicated concentrations of LL-37 alone, LL-37 and human H3, or LL-37 and citrullinated human H3 in minimal medium containing 1 mM magnesium (n=4 for each condition). (D) Growth profiles of *E. coli* treated with 10 µg/mL H2A, 50 µg/mL kanamycin (Kan), or both in minimal medium containing 1 mM magnesium (n=6 for each condition). (E) Scanning electron microscopy (SEM) images of *E. coli* treated with 10 µg/mL H2A and 1 µM LL-37 in minimal medium containing 1 mM magnesium. Blue arrows indicate some of the membrane blebs. (F) Representative phase contrast images and corresponding cell aggregate sizes of *E. coli* that were untreated or treated with 50 µg/mL H2A. *E. coli* were treated with the indicated concentrations of H2A for 1 hour (n=3 for each condition). (G) Representative phase contrast images and corresponding cell sizes of *E. coli* that were untreated or treated with the indicated concentrations of LL-37 for 1 hour (n=3 for each condition). (H) Intracellular propidium iodide (PI) fluorescence intensities of *E. coli* treated with 10 µg/mL H2A, 10 µM magainin-2 (MAG2), or both in minimal medium containing 1 mM magnesium (n=3 for each condition). Points and bars are the average of biologically independent experiments. Error bars indicate standard error of the mean (SEM). One-way ANOVAs were performed. No adjustments were made for multiple comparisons. ns > 0.05. Images are representative of three independent experiments. Scale bars in (B), (E), (F) and (G) represent 5, 5, 3, and 3 µm, respectively.

Supplementary Figure 2

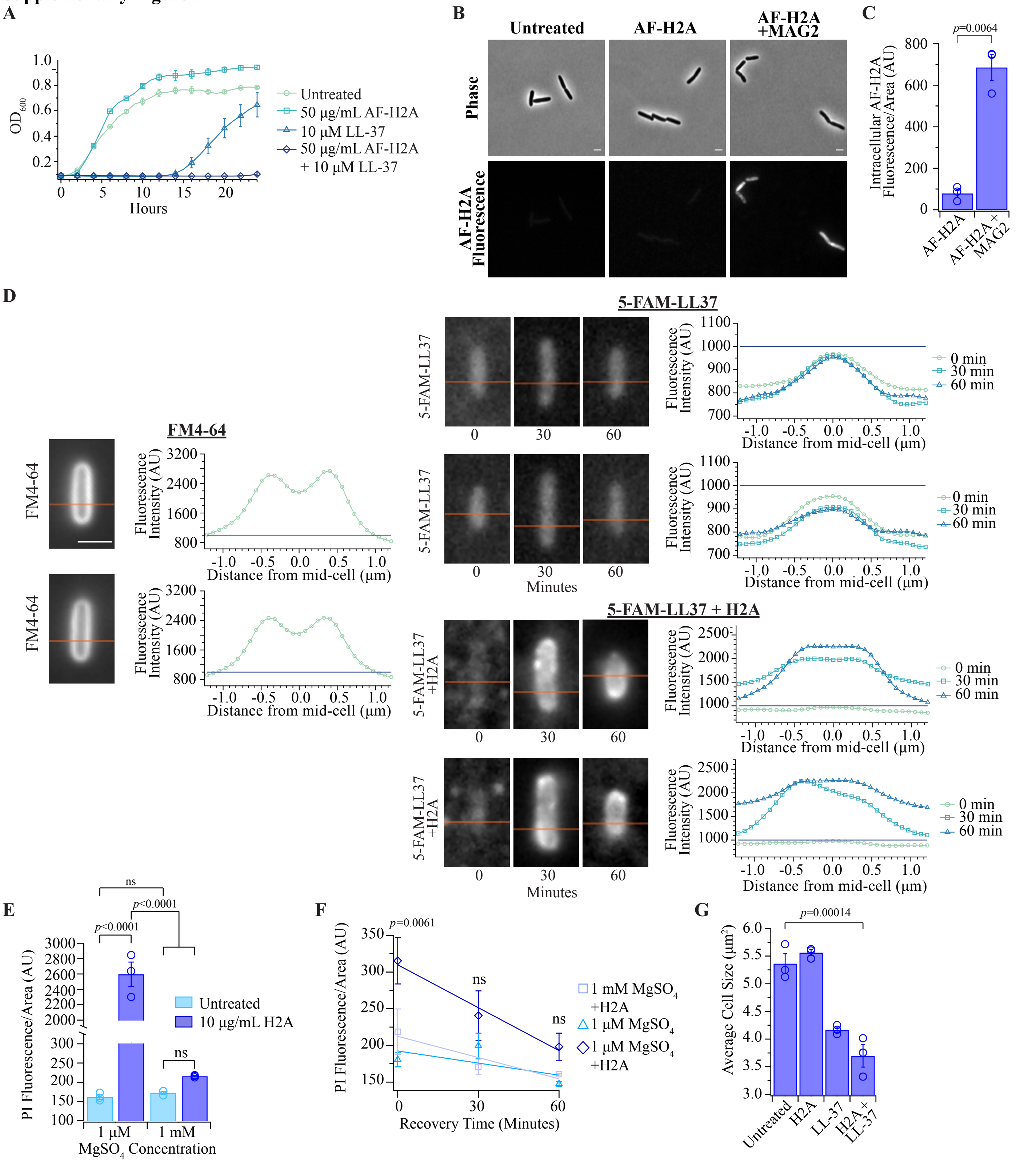

Figure 2. Effects of H2A in com bination with MAG2, recovery from H2A treatment, and the impact of H2A and AMP synergy on cell size. (A) Growth profiles of *E. coli* that were treated with 50 µg/mL fluorescently-labeled H2A (AF-H2A), 10 µM LL-37, or both, in minimal medium containing 1 mM magnesium (n=4 for each condition). (B) Phase contrast, fluorescence images, and (C) intracellular AF-H2A fluorescence intensities of *E. coli* that were untreated or treated with 10 µg/mL AF-H2A alone or in combination with 10 µM MAG2 (n=3 for each condition). AF-H2A is mixed at a concentration of 1% with unlabeled H2A to decrease fluorescence intensity. (D) Representative images and associated fluorescence intensity profiles of *E. coli* that were treated with 1% 5-FAM-LC-LL-37 alone or in combination with 10 µg/mL H2A for 0,30, or 60 minutes. The profiles are taken along the lines indicated in orange. Cell membranes were visualized using FM4-64. (E) Intracellular propidium iodide (PI) fluorescence intensities of H2A-treated *E. coli* in low (1 µM) and physiological (1 mM) magnesium after a 3-hour treatment (n=3 for each condition). (F) Intracellular propidium iodide (PI) fluorescence of *E. coli* over a 1-hour recovery following a 3-hour treatment with 10 µg/mL H2A in minimal medium containing low or physiological magnesium (n=3 for each condition). (G) *E. coli* cell size following 1-hour treatment with 10 µg/mL H2A, 2 µM LL-37, or the combination of H2A and LL-37 in minimal medium containing 1 mM magnesium (n=3 for each condition). Points and bars are the average of biologically independent experiments. Error bars indicate SEM. A one-tailed t-test for C, two-way ANOVAs for E-F, and a one-way ANOVA for G were performed. No adjustments were made for multiple comparisons. ns > 0.05. Images are representative of three independent experiments. Scale bars in represent 2 µm.

Supplementary Figure 3

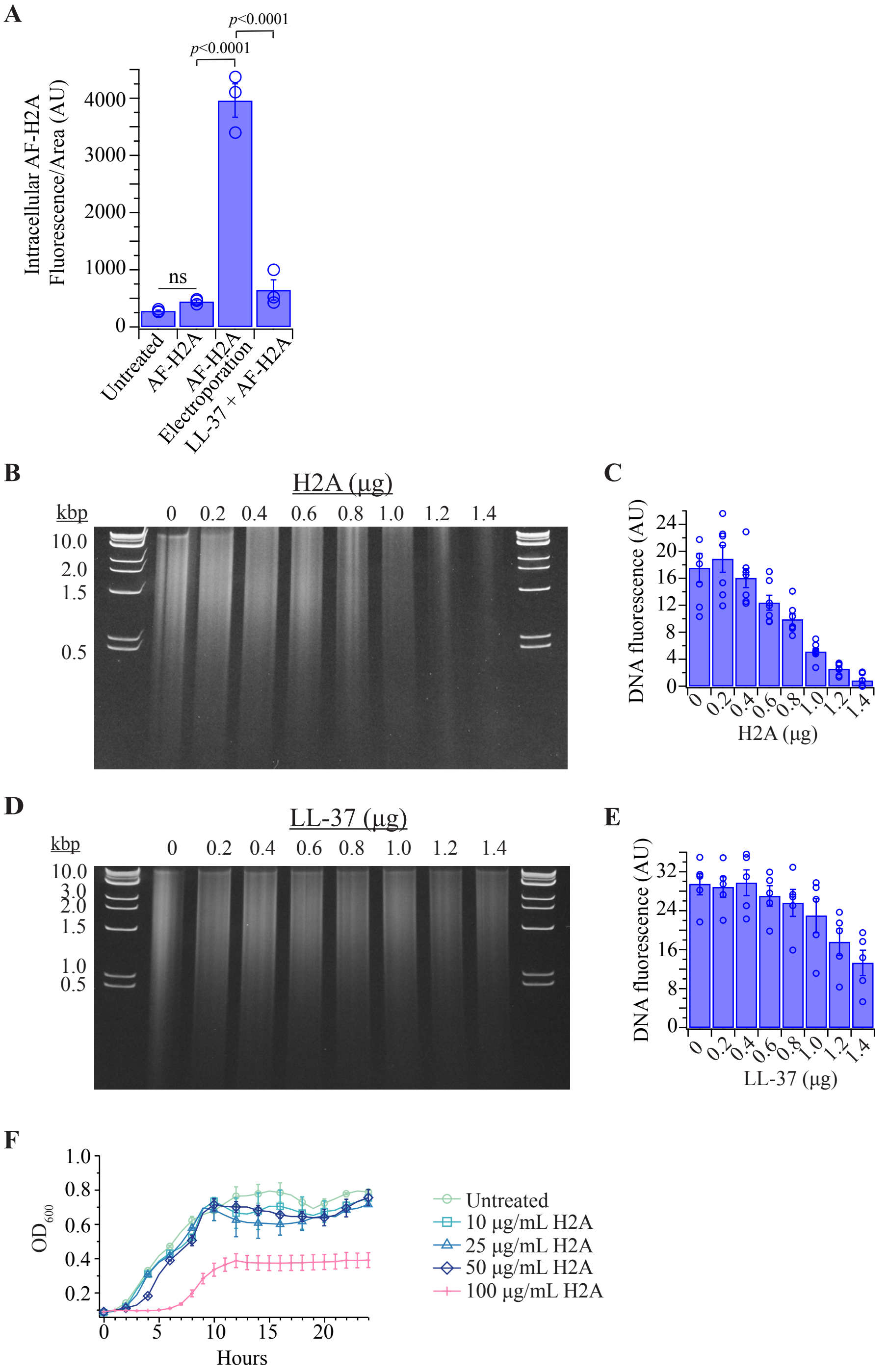

Figure 3. Electroporation facilitates H2A entry into the cytoplasm, H2A inhibits *E. coli* DNA migration, LL-37 has weak interactions with *E. coli* DNA and high concentrations of H2A inhibit *E. coli* growth. (A) AF-H2A fluorescence intensities of *E. coli* that were cultured in the absence of AF-H2A (control), electroporated with 10 μg/mL AF-H2A and cultured in minimal medium containing 1 mM magnesium with the same concentration of AF-H2A, or cultured with 2 μM LL-37 and 10 μg/mL AF-H2A (n=3 for each condition). AF-H2A is mixed with unlabeled H2A (1% AF-H2A, combined concentration of 10 μg/mL). Non-denaturing polyacrylamide gel electrophoresis of 1 μg of *E. coli* DNA mixed with indicated concentrations of (B) H2A or (D) LL-37. Corresponding DNA fluorescence intensities for gels with (C) H2A (n=7) or (E) LL-37 (n=5). (F) Growth profiles of *E. coli* treated with 0, 10, 25, 50, and 100 μg/mL H2A in minimal medium containing 1 mM magnesium (n=4 for each condition). Bars and points are shown as mean ± SEM and are representative of independent experiments. ns > 0.05. A one-way ANOVA was performed for A. No adjustments were made for multiple comparisons. For B-E, DNA was obtained from the same biological sample and gels were processed in parallel.

Supplementary Figure 4

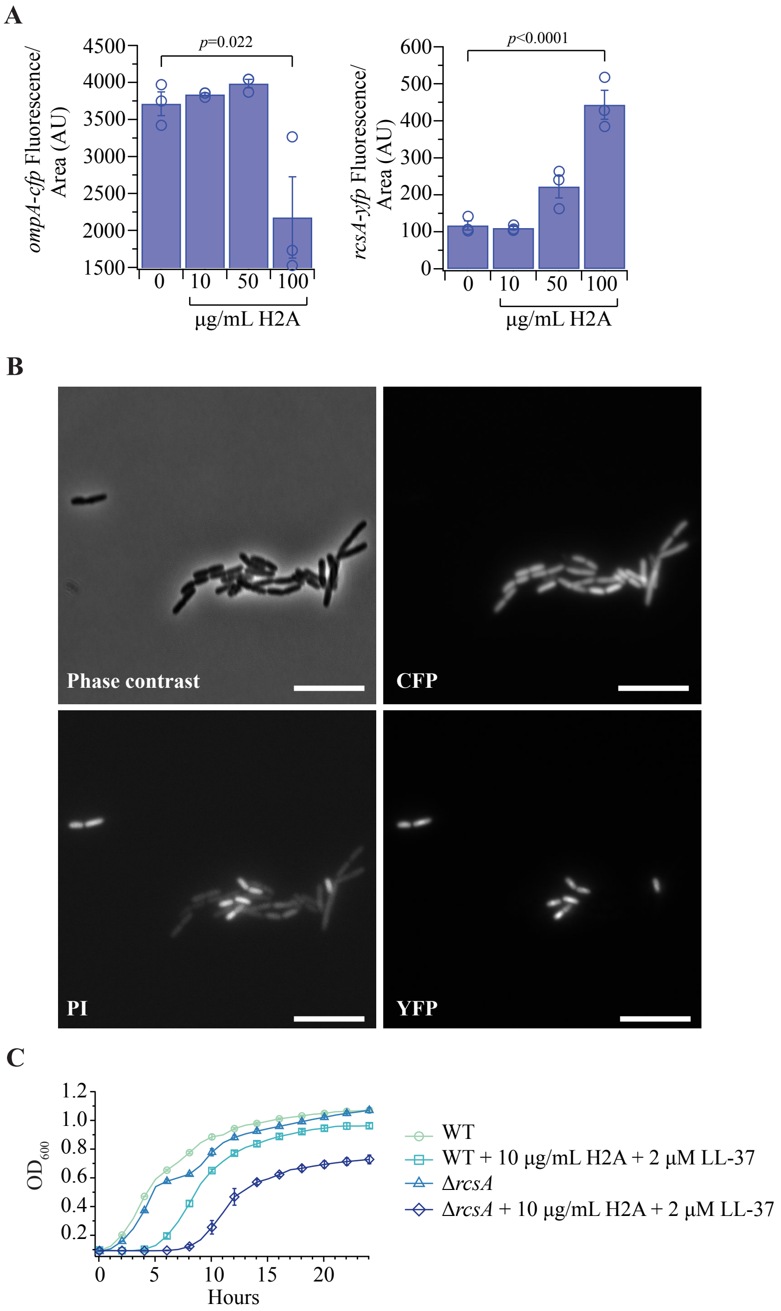

Figure 4. The Rcs phosphorelay pathway is upregulated by histones and improves *E. coli* survival under dual treatment with H2A and LL-37. (A) Intracellular *ompA-cfp* and *rcsA-yfp* fluorescence intensities of *E. coli* after a 1-hour treatment with 0, 10, 50, and 100 µg/mL H2A in medium containing 1 mM magnesium (n=3 for each condition). (B) Phase contrast images and CFP, propidium iodide (PI), and YFP fluorescence images of *E. coli* after a 1-hour treatment with 100 µg/mL H2A (n=3 for each condition). (C) Growth profiles of wild-type *E. coli* and a *rcsA* mutant *E. coli* strain treated with the 10 µg/mL H2A and 2 µM LL-37 (n=4 for each condition). Points and bars are the average of biologically independent experiments. Error bars indicate standard error of the mean (SEM). One-way ANOVAs were performed for A. No adjustments were made for multiple comparisons. Scale bars represent 10 µm.

Supplementary Figure 5

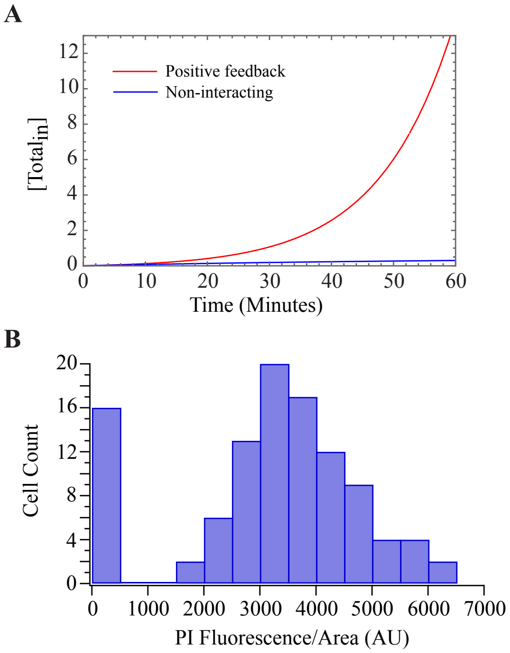

Figure 5. A positive feedback loop underlies histone-AMP synergy. (A) Total intracellular concentration of H2A and AMPs as a function of time for positive feedback (red) and non-interacting (blue) relationships. Positive feedback between H2A and AMPs results in the exponential uptake of H2A and AMPs (red). Removal of the feedback loop results in the uptake at a significantly lower rate (blue). Details of the model and simulation are described in the Supplemental Methods section. (B) Histogram of PI fluorescence intensities in *E. coli* following a 1-hour treatment with 10 μg/mL H2A and 1 μM LL-37 indicates a bimodal distribution of uptake phenotypes.
