# Supplementary material for: Mammalian histones facilitate antimicrobial synergy by disrupting the bacterial proton gradient and chromosome organization
Source: Nat Commun. 2020 Aug 4;11:3888. doi: 10.1038/s41467-020-17699-z (PMC7403156; doi:10.1038/s41467-020-17699-z)
Supplement: Supplementary file 3 — Reporting Summary [file 41467_2020_17699_MOESM3_ESM.pdf]

natureresearch

Corresponding author(s): Steven Gross, Albert Siryaporn

Last updated by author(s): ~~Apr 27,~~ 2020

Reporting Summary

Nature Research wishes to improve the reproducibility of the work that we publish. This form provides structure for consistency and transparency in reporting. For further information on Nature Research policies, see Authors & Referees and the Editorial Policy Checklist.

Statistics

For all statistical analyses, confirm that the following items are present in the figure legend, table legend, main text, or Methods section.

n/a	Confirmed	
☐	☒	The exact sample size (*n*) for each experimental group/condition, given as a discrete number and unit of measurement
☐	☒	A statement on whether measurements were taken from distinct samples or whether the same sample was measured repeatedly
☐	☒	The statistical test(s) used AND whether they are one- or two-sided<br>*Only common tests should be described solely by name; describe more complex techniques in the Methods section.*
☐	☒	A description of all covariates tested
☐	☒	A description of any assumptions or corrections, such as tests of normality and adjustment for multiple comparisons
☐	☒	A full description of the statistical parameters including central tendency (e.g. means) or other basic estimates (e.g. regression coefficient) AND variation (e.g. standard deviation) or associated estimates of uncertainty (e.g. confidence intervals)
☐	☒	For null hypothesis testing, the test statistic (e.g. *F*, *t*, *r*) with confidence intervals, effect sizes, degrees of freedom and *P* value noted<br>*Give P values as exact values whenever suitable.*
☒	☐	For Bayesian analysis, information on the choice of priors and Markov chain Monte Carlo settings
☒	☐	For hierarchical and complex designs, identification of the appropriate level for tests and full reporting of outcomes
☒	☐	Estimates of effect sizes (e.g. Cohen's *d*, Pearson's *r*), indicating how they were calculated

Our web collection on statistics for biologists contains articles on many of the points above.

Software and code

Policy information about availability of computer code

Data collection	NIS Elements v. 4.5 was used to acquire microscopy images.
Data analysis	Matlab v2015, v2017, R v.3.4.3, Image J v1.51k, Micros~~copy~~ Excel, and our custom-written Matlab scripts were used for analysis.

For manuscripts utilizing custom algorithms or software that are central to the research but not yet described in published literature, software must be made available to editors/reviewers. We strongly encourage code deposition in a community repository (e.g. GitHub). See the Nature Research guidelines for submitting code & software for further information.

Data

Policy information about availability of data

All manuscripts must include a data availability statement. This statement should provide the following information, where applicable:

- Accession codes, unique identifiers, or web links for publicly available datasets
- A list of figures that have associated raw data
- A description of any restrictions on data availability

Raw data for Figures 1C,1E,1G; 2B,2D,2F,2H; 3B-F; 4B,4C,4H-J; S1A,S1F-H; S2C,S2E-G; S3A are available in the Supplementary Information. The RNA-Seq data is freely available under the National Center for Biotechnology Information Gene Expression Omnibus (accession number GSE142755 (temporary token for reviewers: uxghcgcotlafhuf)). Additional raw data that support the findings of this study are available from the corresponding authors upon request.

Field-specific reporting

Please select the one below that is the best fit for your research. If you are not sure, read the appropriate sections before making your selection.

☒ Life sciences ☐ Behavioural & social sciences ☐ Ecological, evolutionary & environmental sciences

For a reference copy of the document with all sections, see nature.com/documents/nr-reporting-summary-flat.pdf

Life sciences study design

All studies must disclose on these points even when the disclosure is negative.

ample size	For each experimental condition, measurements were performed on at least 3 independent biological replicates. For microscopy experiments, at least 100 cells were analyzed for each replicate. These sample sizes were chosen to provide measurements of variance for each experimental condition such that statistical significance could be assessed.
ata exclusions	~~Replicate data were rejected using pre-established criteria for values that were more than 3 sigma from the mean.~~
eplication	Findings were replicated by performing multiple independent biological experiments.
andomization	Sample measurements were randomized when possible by renaming samples to a descriptor that excluded details of the sample. Measurement order was randomized when possible.
inding	Samples measurements were blinded when possible by renaming samples to a descriptor that excluded details of the sample.

Reporting for specific materials, systems and methods

We require information from authors about some types of materials, experimental systems and methods used in many studies. Here, indicate whether each material, system or method listed is relevant to your study. If you are not sure if a list item applies to your research, read the appropriate section before selecting a response.

Materials & experimental systems

n/a	Involved in the study	
☒	☐	Antibodies
☒	☐	Eukaryotic cell lines
☒	☐	Palaeontology
☒	☐	Animals and other organisms
☒	☐	Human research participants
☒	☐	Clinical data

Methods

n/a	Involved in the study	
☒	☐	ChIP-seq
☒	☐	Flow cytometry
☒	☐	MRI-based neuroimaging
